# Characterization of TrxC, an Atypical Thioredoxin Exclusively Present in Cyanobacteria

**DOI:** 10.3390/antiox7110164

**Published:** 2018-11-13

**Authors:** Luis López-Maury, Luis G. Heredia-Martínez, Francisco J. Florencio

**Affiliations:** Instituto de Bioquímica Vegetal y Fotosíntesis, Universidad de Sevilla-CSIC, Av/Américo Vespucio 49, E-41092 Sevilla, Spain; heredia@ibvf.csic.es (L.G.H.-M.); floren@us.es (F.J.F.)

**Keywords:** cyanobacteria, thioredoxin, photosynthesis

## Abstract

Cyanobacteria form a diverse group of oxygenic photosynthetic prokaryotes considered to be the antecessor of plant chloroplast. They contain four different thioredoxins isoforms, three of them corresponding to *m*, *x* and *y* type present in plant chloroplast, while the fourth one (named TrxC) is exclusively found in cyanobacteria. TrxC has a modified active site (WCGLC) instead of the canonical (WCGPC) present in most thioredoxins. We have purified it and assayed its activity but surprisingly TrxC lacked all the classical activities, such as insulin precipitation or activation of the fructose-1,6-bisphosphatase. Mutants lacking *trxC* or over-expressing it were generated in the model cyanobacterium *Synechocystis* sp. PCC 6803 and their phenotypes have been analyzed. The *ΔtrxC* mutant grew at similar rates to WT in all conditions tested although it showed an increased carotenoid content especially under low carbon conditions. Overexpression strains showed reduced growth under the same conditions and accumulated lower amounts of carotenoids. They also showed lower oxygen evolution rates at high light but higher Fv’/Fm’ and Non-photochemical-quenching (NPQ) in dark adapted cells, suggesting a more oxidized plastoquinone pool. All these data suggest that TrxC might have a role in regulating photosynthetic adaptation to low carbon and/or high light conditions.

## 1. Introduction

Thioredoxin are small (~12 kDa) evolutionary conserved proteins with redox activity that present a conserved three-dimensional structure denominated “Trx fold”, composed of five β strands surrounded by four α helices [1]. They serve as redox carriers and catalyse reduction of other proteins activating/deactivating them. Thioredoxins contain a conserved disulphide active site in the form WCGPC that undergoes oxidation-reduction cycles. Most organisms contain at least one thioredoxin gene although their numbers are expanded in photosynthetic organisms [2,3]. *Arabidopsis* contains at least twenty thioredoxin isoforms, ten are present in *Chlamydomonas reinhardtii* in contrast to humans or *Escherichia coli* that only contain two. *Arabidopsis* thioredoxins are classified in seven groups (Trx *h*, Trx *o*, Trx *f*, Trx *z*, Trx *m*, Trx *x* and Trx *y*) and all of them are also present in *Chlamydomonas*. Of these, Trx *m*, Trx *x* and Trx *y* have a cyanobacterial origin, and together with Trx *f* and Trx *z* are located to the chloroplast [4,5]. In cyanobacteria, four thioredoxins classes have been found along the genome of more than 300 species, three of them corresponding to *m*, *x* and *y* types present in plant chloroplast, while the fourth one is exclusively found in cyanobacteria (TrxC) [6].

Thioredoxins are reduced by thioredoxin reductases of which at least two different families exist: NADPH-dependent thioredoxin reductases (NTR) and ferredoxin-dependent thioredoxin reductases (FTR). NTR are present from bacteria to humans while FTR is an Fe-S protein that uses ferredoxin and is present in photosynthetic organisms [2]. Thioredoxin reduction system is also diverse in cyanobacteria, most of them containing the FTR system (except *Gloeobacter* and *Prochlorococcus* groups) [2,6]. Some cyanobacteria also contain NTRC, a protein that contains a NTR module fused to a thioredoxin module in a single polipeptide, which seems to function as a reducing system for the 2-cys peroxiredoxin in *Anabaena* sp. PCC 7120 (hereafter *Anabaena*) [7,8,9]. They can also contain two other NTR related proteins that were initially annotated as NADPH dependent enzymes. However, these two proteins have recently been shown not to use or bind NADPH, as they lack the aminoacid signature characteristic of the NADPH binding domain, and have been renamed DTR (for Deeply rooted bacterial Thioredoxin Reductase) and DDOR (Diflavin-linked Disulfide OxidoReductase) [10,11]. Dithionite reduced DTR is able to reduce thioredoxins although its physiological reductant is still unknown [10]. In contrast, DDOR shows a new structure containing two to Flavin Adenine Dinucleotide (FAD) per monomer, does not reduce thioredoxins and probably functions as an oxidase [11]. These proteins are scattered distributed in cyanobacteria but strains lacking FTR usually contain a gene coding for DTR. Of these two proteins, only DDOR is present in *Synechocystis* sp. PCC 6803 (hereafter *Synechocystis*) although some cyanobacteria (such as *Gloeobacter violaceus* sp. PCC 7421) contains both [2,10,11].

Putative thioredoxins targets have been studied in cyanobacteria by several proteomics approaches [12,13,14,15,16,17]. A high degree of overlapping targets for the different isoforms have been found, highlighting that these in vitro proteomics approaches identify reactive cysteine that could be subjected to redox regulation in vivo and that overlapping and redundant roles are expected for the different thioredoxins in vivo. The role of the different thioredoxins has been also analyzed by generating mutants in their corresponding genes. Trx *m* (*trxA*) has been shown to be essential in unicellular cyanobacteria and therefore no specific phenotypes have been associated to it [18,19]. More recently, mutants in *trxM* genes have been described in *Anabaena* which possess two genes coding for Trx *m*: *trxM1* and *trxM2*. Both genes are dispensable although *trxM1* mutant showed a pleiotropic phenotype and was unable to grow in diazotrophic conditions, while *trxM2* lack any appreciable phenotype [20,21]. In plants *trxM* are involved in several functions related to fluctuating light conditions, cyclic electron flow or meristem maintenance [4,5,22]. In *Synechocystis trxB*^−^ mutant strains (lacking *x* type) showed sensitivity towards HL and to the presence of DTT in the culture media [23], while *trxQ*^−^ mutant (lacking *y* type) showed sensitivity to oxidative stress induced by methyl viologen [23]. All three proteins were able to interact and reduce the different peroxiredoxins present in *Synechocystis* although with different efficiencies, with TrxQ being the most efficient in vitro [24]. The fourth group, TrxC, is only found in cyanobacteria. No activity or function has been ascribed to this class of Trx in *Synechocystis* as mutants lacking it did not show any phenotype [25], although *Anabaena trxC*^−^ mutant showed more oxidative stress than WT in nitrate grown cultures [20]. Furthermore, *Anabaena* TrxC seems to be inactive in reducing OpcA or G6PDH [21]. Here we describe the characterization of *Synechocystis*’ TrxC protein and of mutants either lacking *trxC* or overexpressing it. 

## 2. Materials and Methods

### 2.1. Strains and Culture Conditions

*Synechocystis* cells were grown photoautotrophically on BG11 or BG11C [26] at 30 °C under continuous illumination (50 to 180 µmol photons m^−2^ s^−1^) and bubbled with a stream of 1% (*v*/*v*) CO_2_ in air as indicated. BG11 media was buffered with 10 mM TES-NaOH pH 7.5. For plate cultures, medium was supplemented with 1% (*wt*/*vol*) agar. All media contained the standard copper concentration (0.3 µM) except in Figure 2 in which BG11-Cu was used. Kanamycin and nourseothricin, were added to a final concentration of 50 µg mL^−1^. *Synechocystis* strains and their relevant genotypes are described in Table 1. *E. coli* DH5α or BL21 cells were grown in Luria broth medium and supplemented with 100 µg mL^−1^ ampicillin, 50 µg mL^−1^ kanamycin and 50 µg mL^−1^ nourseothricin when required. 

### 2.2. Western Blotting

Crude extracts were prepared using glass beads and vigorous vortexing using a minibead-beater. Cells (corresponding to 20 OD_750nm_) were resuspended in 300 µL of buffer A (50 mM Tris-HCl pH 8.0, 50 mM NaCl) and subjected to 2 cycles of 1 min vortexing separated by 5 min of cooling on ice. Cell extracts were recovered from the beads by piercing a hole in bottom of the tube and samples were clarified by two sequential centrifugations: 5’ at 3000× *g* to eliminate cells debris and twice 15 min at 18,000× *g* to collect membranes. Protein concentration in cell-free extracts by the method of Lowry, using Bovine Serum Albumin as a standard and the specified amounts of proteins were separated on sodium dodecyl sulfate polyacrylamide gel electrophoresis (SDS-PAGE). Gels were transferred to nitrocellulose membranes (Bio-Rad, Düsseldorf, Germany catalog #162-0115), blocked in phosphate-buffered saline (PBS) containing 0.1% Tween 20 and 5% of skimmed milk and incubated with antibodies against TrxA (1:3000), TrxB (1:3000), TrxQ (1:2000), TrxC (1:1000), DDOR (1:5000), GrxA (1:3000), GrxC (1:3000) and 2cysprx (sll0755; 1:5000). The ECL Prime immunoblotting system (GE Healthcare, Little Chalfont) was used to detect the different antigens with goat anti-rabbit conjugated to horseradish peroxidase (Sigma St Louis, USA catalog #A6154) diluted to 1:25,000. 

### 2.3. Cloning and Purification of TrxC

*TrxC* gene was cloned from *Synechocystis*’ DNA after PCR amplification with oligonucleotides Syn_TrxC_BamHI_NdeI and SynTrxC_R_SalI and cloned into NdeI-SalI digested pET28 or *Bam*HI-*Sal*I digested pGEX6P to generate pET_trxC or pGST_trxC respectively. To generate site directed TrxCL32P mutant a 366 pb fragment was amplified by two-step PCR using oligonucleotides TrxC_NdeI-trxC_L32P_Rv and trxC_L32P_Fw-TrxC_NotI, that introduced the desired mutation, and cloned into pGEMT generating pG_TrxCL32P. The plasmid was cut with *Nde*I-*Not*I and a 347 pb fragment was ligated to NdeI-Not digested pET28 to generate pET_TrxCL32P. Sequence of all oligonucleotides are provided in Table S1.

TrxC fusion proteins were expressed in *E. coli* BL21. 200 mL of culture was grown in Luria broth medium to an optical density at 600 nm of 0.6, cooled to 4 °C, induced with 0.5 mM isopropyl-β-d-thiogalactopyranoside for 2.5 h and harvested by centrifugation. For his tagged TrxC purification, cells were resuspended in 50 mM Tris-HCl pH 8.0, 500 mM NaCl, 1 mM PMSF and disrupted (20 kHz, 75 W) on ice for 3 min (in 30-s periods) in a Branson sonicator. Lysates were clarified by centrifugation 20,000× *g* for 30 min. Supernatants were supplemented with imidazole to a final concentration of 25 mM and loaded onto a 2 mL Ni-NTA agarose (IBA, Goettingen, Germany catalog #2-3201-025) column for affinity chromatography purification. Column was washed with 50 mM Tris-HCl pH 8.0, 500 mM NaCl, 25 mM imidazole until no protein was detected and bound recombinant proteins were eluted with 250 mM of imidazole in 50 mM Tris-HCl pH 8.0, 500 mM NaCl. For GST and GST-TrxC purification cell pellets were resuspended in 5 mL of PBS buffer (150 mM NaCl, 16 mM Na_2_HPO_4_, 4 mM NaH_2_PO_4_, 1 mM phenylmethylsulfonyl fluoride, 7 mM β-mercaptoethanol) supplemented with 0.1% Triton X-100. Cells extract were prepared as above, mixed with 1 mL of glutathione agarose beads (GE Healthcare, Little Chalfont catalog #17-0756-01) and incubated for 2 h at 4 °C with gentle agitation. Then beads were transferred to a column and washed extensively with PBS buffer until no more protein was eluted from the column. GST and GST-TrxC were eluted in 50 mM Tris HCl pH 8.0, 10 mM GSH. TrxC was excised by digestion with PreScission Protease (GE Healthcare, Little Chalfont catalog #27084301) following manufacturer’s instructions.

### 2.4. Mutant Construction

To generate site STXC2 mutant, a 1170 pb fragment was amplified using oligonucleotides trxCHIII and trxCXhoI and cloned into pBS digested with the same enzymes generating pSTXC1. A CK1 cassette was cloned into pSTXC1 digested with *Hinc*II (deleting 345 pb and expanding the whole ORF) generating pSTXC2. This plasmid was used to transform *Synechocystis* generating STXC2 strain. For the overexpression strains a *Xba*I-*Not*I fragment from pET_trxC was cloned into pN:PpetE:Nat (a plasmid containing *glnN* gene in which the *petE* regulatory region, a multiple cloning site and a Nourseothricin resistance cassette has been cloned [27]) digested in the same way generating pNPpetEtrxC. This plasmid was used to transform both WT and STXC2 generating WTOE and STXCOE strains (Table 1).

### 2.5. FBPase Activation Assay

Recombinant pea FBPase was purified by Ni nickel-affinity column as previously described [28] and the eluted protein was desalted with a PD10 column in 50 mM Tris HCl pH 8.0, 150mM NaCl. 2 µg of recombinant pea FBPase was incubated with DTT (10 mM or 0.1 mM) and 3–30 µg of thioredoxins for 10 min in 175 µL containing 30 mM Tris·HCl pH 8.0, 7 mM MgCl_2_ at 30 °C. Then, 25 µL of fructose-1,6-bisphosphate 50 mM was added and the reaction was incubated for 30 min at 30 °C. Also, 1 mL LPi mix (2.5% sulfuric acid, 7.5 mM ammonium heptamolybdate, 100 mM FeSO_4_) was added to stop the reaction and the Pi released was measured at 660 nm [29]. A calibration curve with known concentrations of Na_2_PO_4_ was used to calculate Pi concentration.

### 2.6. Insulin Reduction Assay

Thioredoxin insulin reduction assay was carried out using 3 μg of recombinant HisTrxA or HisTrxC in 1 mL of 0.1 M potassium phosphate pH 7.0, 2 mM EDTA, 1 mM DTT and 1mg/ml insulin at 30 °C. The same buffer without DTT was used as reference. Thioredoxin activity was measured by the increase of turbidity at OD_650nm_ due to insulin precipitation [30]. Recombinant HisTrxA was expressed and purified as described in [13,14].

### 2.7. PAM

A pulse amplitude modulated fluorometer DUAL-PAM-100 (Walz, Effeltrich, Germany) was used to monitor chlorophyll, a fluorescence in intact cells adjusted to OD_750nm_ = 1. Measurements were performed in 1 cm × 1 cm cuvettes at 30 °C. Red (620 nm) actinic lights was used as background light and saturating pulses (10,000 µmol photons m^−2^ s^−1^, 635 nm, 300 ms) were applied to transiently close all PSII centers. The maximal photochemical efficiency of PSII (Fv/Fm) was measured in the presence of 3-(3,4-dichlorophenyl)-1,1-dimethylurea (DCMU). Fm’_dark_ is defined as the Fm of cells adapted to dark conditions for 10 min. Non-photochemical-quenching (NPQ) was calculated as Fm-Fm’_dark_/Fm’_dark_ as described in [31]. 

### 2.8. Oxygen Evolution

Oxygen evolution was measured in Clark-type oxygen electrode (Hansatech Chlorolab 2) using mid-logarithmic (OD_750nm_ = 0.8–1) cultures adjusted to OD_750nm_ = 0.5 in BG11 pH 7.5 media supplemented with 20 mM NaHCO_3_ using white LED light. 

## 3. Results

### 3.1. TrxC Is an Atypical Thioredoxin Exclusively Found in Cyanobacteria

**Figure 1:**
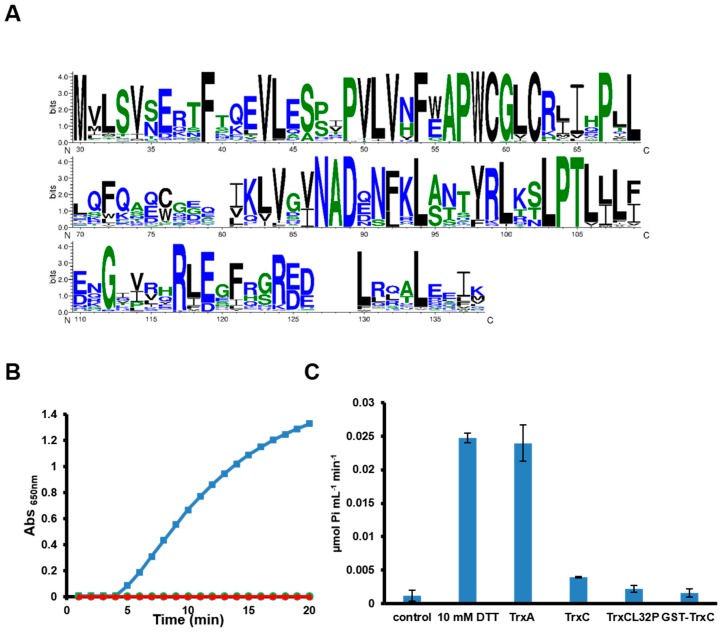
TrxC is an atypical thioredoxin. (**A**) Sequence logo of TrxC proteins form cyanobacteria. TrxC proteins were identified using blast at NCBI and manually curated to retain only those with a WCGL/V/IC sequence (269 sequences). This sequences were aligned using muscle and the alignment was submitted to weblogo3 to generate the consensus sequence shown. (**B**) Insulin reduction assay. 3 µM of recombinant TrxA (■), TrxC (●) and TrxCL32P (▲) were incubated with insulin in the presence of 1 mM of DTT. Insulin precipitation was measured as an increase in absorbance at 650 nm. Three independent purification were assayed for TrxC and two for TrxCL32P with identical results to the one shown. (**C**) FBPase activation assay. Oxidased pea FBPase was preincubated for 30 min with 100 µM DTT (control), 10 mM DTT or 100 µM DTT and 3 µM of TrxA, TrxC, TrxCL32P or 30 µM GST-TrxC. Data are the mean and standard error of 3 independent assays.

Of the four thioredoxins found in cyanobacteria, only TrxC is not homologous to thioredoxins present in plant chloroplasts. Furthermore, analysis of its sequence shows an altered catalytic site WCGL/V/IC that is highly conserved in cyanobacteria (Figure 1A). Despite the N terminal part of the sequence being similar to other Trx, most of the protein shows a clear divergence from other thioredoxin in cyanobacteria and less conservation in the C-terminal sequence of the protein (Figure 1A). There is no other thioredoxin sequence with a similar active site in any other organism. Moreover, all cyanobacterial groups except *Prochlorococcus* and *Gloeobacteria* contain at least one species that presents a *trxC* gene (Table S2) existing in more than 200 strains. This distribution suggests that this protein may have an important role in cyanobacterial environmental adaptation or metabolism. 

In order to characterize TrxC function we have produced it as recombinant His-tagged version in *E. coli*. The protein was expressed at high levels although it was difficult to purify as it was mostly in the insoluble fraction, probably forming inclusion bodies. Only low amounts were recovered in the soluble fraction and this protein was prone to precipitation when concentrated, but we purified enough protein to assay its activity. In contrast to TrxA (*m*-type), TrxC did not reduce insulin in the presence of DTT (Figure 1B). To further characterize the protein, we analyzed chloroplast pea FBPase activation by TrxC. In the same way as in the insulin reduction assay, TrxA was able to activate chloroplast FBPase, while TrxC did not activate it. As TrxC presents an unconventional active site WCGLC, this was changed to a canonical Trx active site (WGCPC) by site directed mutagenesis generating TrxCL32P. The protein was purified and used in both assays with identical results to the WT protein (Figure 1B,C). To further confirm these results we generate a recombinant version of the protein fussed to GST (GST-TrxC), which allowed us to purify higher amounts of soluble recombinant protein. Although TrxC can be excised from GST by protease digestion, the TrxC portion precipitated rapidly after digestion and therefore only GST-TrxC was used. GST and GST-TrxC were used to assay chloroplast FBPase activation at higher concentrations (30 µM) but neither of the proteins activated FBPase. These data suggest that TrxC is not active in the classical thioredoxin assays and that this is not related to its unconventional active site. 

### 3.2. TrxC Mutant and Overexpression Strains Characterization

**Figure 2:**
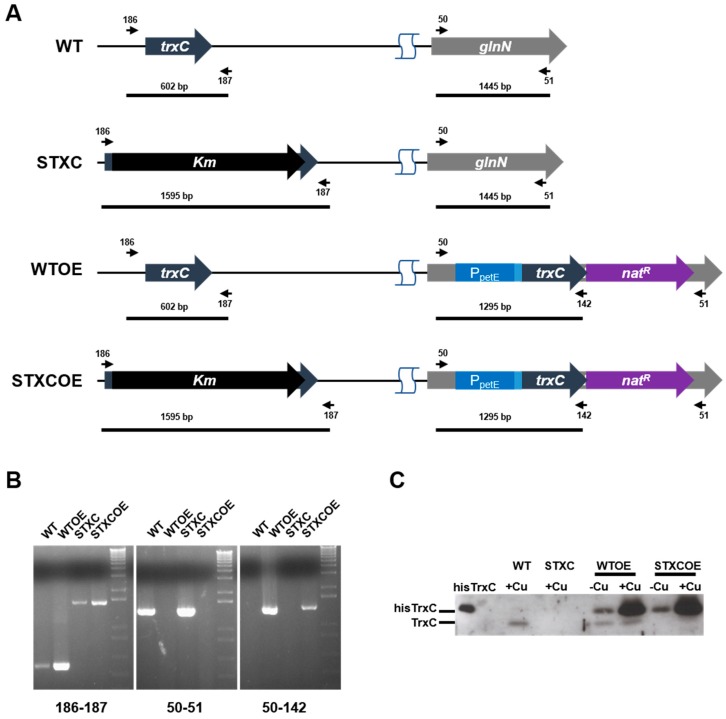
Construction of WT, WT_OE, STXC and STXCOE strains. (**A**) Schematic representation of the *trxC* and *glnN loci* in the WT and mutant strains. (**B**) PCR analysis of the mutant strains using the oligonucleotides indicated in (A). (**C**) Western-blot analysis of TrxC protein levels in the WT, WTOE, STXC and STXCOE strains. Cells were grown in BG11 supplemented with 1% CO_2_ to mid-log growth phase and 1 OD_750nm_ was collected before and after 2 h of 0.3 µM Cu addition. The pellet was resuspended in 1× Laemmli buffer and boiled for 10 min, then 20 µL of the boiled cell suspension were loaded on and gel and analyzed by western blot.

To investigate TrxC physiological function, a mutant lacking *trxC* (STXC2 mutant strain) and mutants over-expressing a His-tagged version of the protein (HisTrxC) were constructed. For overexpression, the His-tagged *trxC* gene was placed under control of the copper inducible *petE* promoter in a WT and STXC2 backgrounds, generating WTOE and STXCOE strains, respectively (Table 1). All mutants were verified by PCR analysis and shown to be completely segregated (Figure 2A,B). All strains presented similar growth rates to the WT in BG11C plates and liquid media under our standard growth conditions ([25]; data not shown). TrxC protein levels were analyzed in the mutants by western blot using TrxC antibodies in whole cell extracts. TrxC was detected in WT and WTOE strains but not in STXC2 and STXCOE, while a band corresponding to the recombinant HisTrxC was detected in both WTOE and STXCOE strains. Furthermore, HisTrxC expression was regulated by the presence of copper in these strains, the amount of HisTrxC increased after copper addition in both strains. Surprisingly HisTrxC expression levels were higher than endogenous TrxC levels in the WT strain even in the absence of copper in the media in both WTOE and STXCOE strains and was further elevated in the presence of copper (Figure 2C).

**Figure 3:**
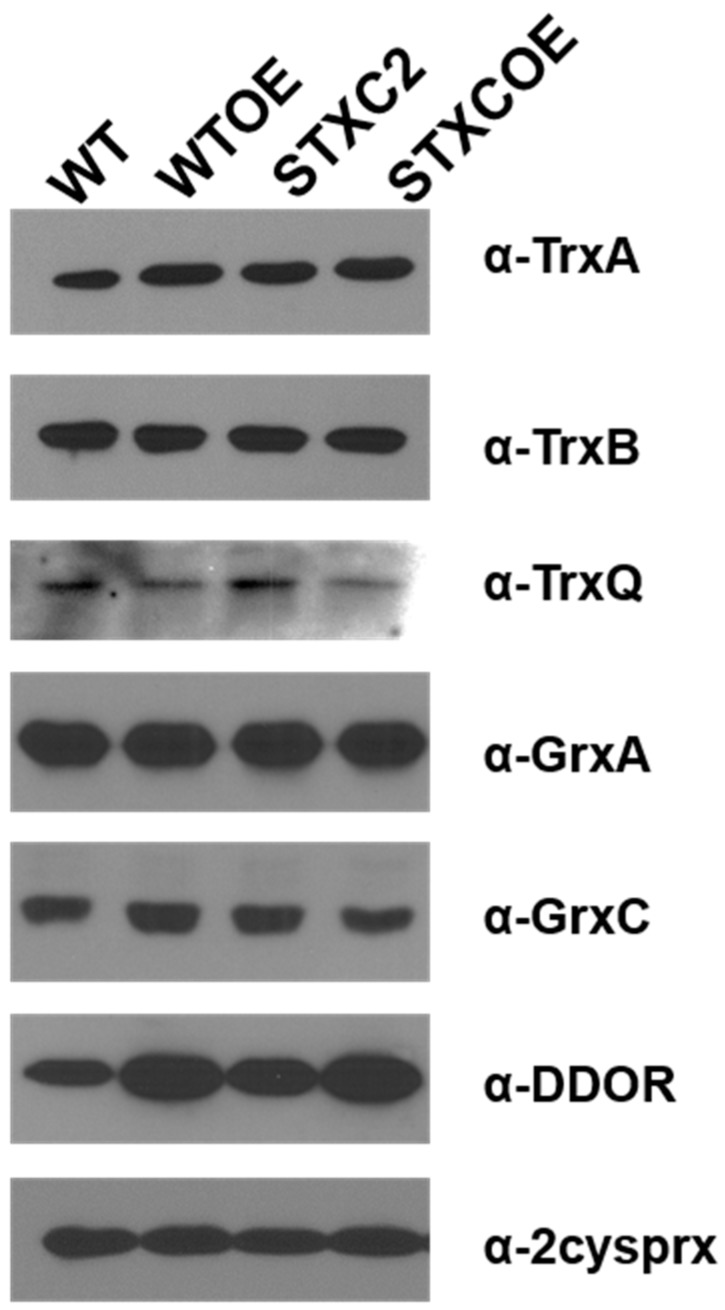
Redox related proteins in WT, WTOE, STXC2 and STXCOE strains. Western-blot analysis of TrxA, TrxB, TrxQ, GrxA, GrxC, DDOR and 2-cysprx in the WT, WTOE, STXC2 and STXCOE strains. Cells were grown in BG11 supplemented with 1% CO_2_ to mid-log growth phase and collected. Cells were broken and 20 μg of total protein from soluble extracts were separated by SDS PAGE and analysed by western blot to detect the different proteins using specific antibodies. Experiments were repeated at least two (for TrxQ antibody) or three (all other antibodies) times with biological independent samples. SDS PAGE: sodium dodecyl sulfate polyacrylamide gel electrophoresis.

To study the TrxC impact on redox regulation in *Synechocystis*, we have analyzed the expression levels of other redox related proteins in WT and *trxC* mutant strains. All strains were grown to exponential phase in copper containing media and levels of different proteins were analyzed by western blot. Levels of the other thioredoxins (TrxA, TrxB and TrxQ), glutaredoxins (GrxA and GrxC) and 2-cys peroxiredoxin did not change in the mutants (Figure 3). In contrast, levels of DDOR (*slr0600*) increased in both the WTOE y STXCOE strains (Figure 3) in which TrxC levels were also increased (Figure 2C). These data showed that TrxC did not affect levels of other thioredoxins and glutaredoxins. The changes in DDOR protein levels suggest that these two proteins could be functionally related. 

### 3.3. Growth of TrxC Mutant Strains under Low CO_2_ Conditions

**Figure 4:**
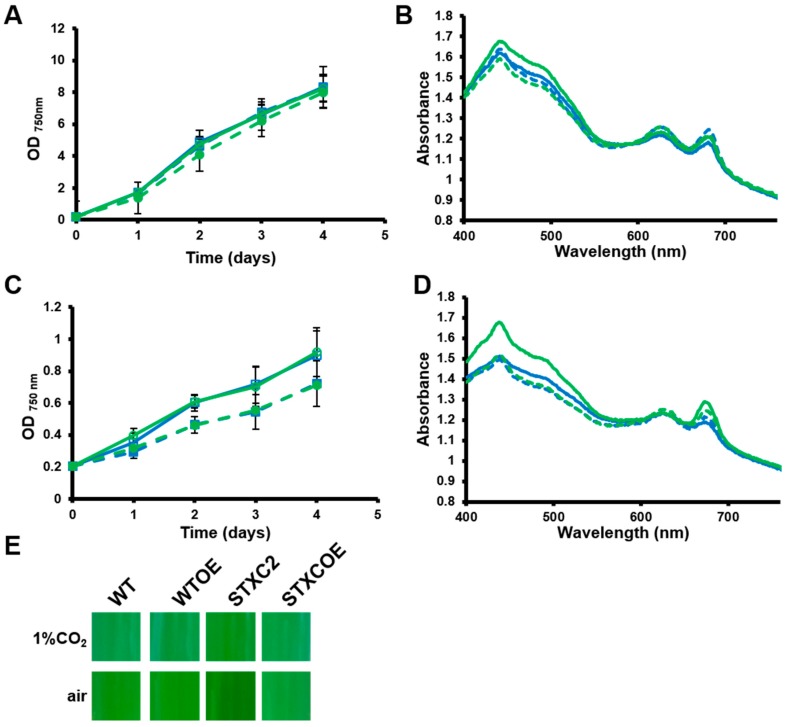
Overexpression of *trxC* slows growth in low carbon conditions. (**A**) Growth of *trxC* mutant strains in high carbon conditions. WT (□), STXC2 (○), WTOE (■) and STXCOE (●) were grown in BG11 pH 7.5 under low light until the exponential phase, diluted to 0.2 OD_750nm_ and shifted to 180 μmol m^−2^ s^−1^ light intensity bubbled with air + 1% CO_2_. Growth was monitored by measuring OD_750nm_. Data represented are the mean and standard error of 3–4 (depending on the time point) biological independent cultures. (**B**) Whole cell spectra of WT (blue solid line), STXC2 (green solid line), WTOE (blue dashed line) and STXCOE (green dashed line) grown as in (A). (**C**) Growth of *trxC* mutant strains in low carbon conditions. WT (□), STXC2 (○), WTOE (■) and STXCOE (●) were grown in BG11 pH 7.5 under low light until the exponential phase, diluted to 0.2 OD_750nm_ and shifted to 180 μmol m^−2^ s^−1^ light intensity and bubbled with air. Growth was monitored by measuring OD_750nm_. Data represented are the mean and standard error of 3–4 (depending on the time point) biological independent cultures. (**D**) Whole cell spectra of WT (blue solid line), STXC2 (green solid line), WTOE (blue dashed line) and STXCOE (green dashed line) grown as in (C). (**E**) Photograph of WT, STXC2, WTOE and STXCOE cultures grown in BG11 pH 7.5 bubbled with air + 1% CO_2_ or air.

In order to investigate the physiological role of TrxC, *trxC* mutant strains were analyzed under several growth conditions. No difference between strains was detected in BG11C (containing NaHCO_3_). We have only detected differences in growth and/or pigmentation under moderate light intensities and low carbon conditions (BG11 pH 7.5 bubble with air + 1% CO_2_ or BG11 pH 7.5 bubbled with air). We have selected an intermediate light intensity (180 µmol m^−2^ s^−1^) and air bubbled cultures (low carbon availability) as these were the most consistent growth conditions in which we were able to detect differences. At higher light intensities (500 µmol m^−2^ s^−1^), even the WT strain showed impaired growth and in some cases died. All strains were previously adapted to BG11 pH 7.5 and high carbon (bubbled with air + 1% CO_2_) in low light (50 µmol m^−2^ s^−1^) until they reached late exponential phase (OD_750nm_ = 1–2) and then were diluted to OD_750nm_ = 0.2 and shifted 180 µmol m^−2^ s^−1^ in either high carbon (air + 1% CO_2_) or low carbon conditions (air); this adaptation step was necessary for the strains to show consistent growth under this condition. In cultures bubbled with air + 1% CO_2_ all strains grew at similar rates but differences in color were observed (Figure 4A). These are clearly visible in whole cell absorption spectra in which STXC2 showed a higher absorption at 485 nm, which corresponds to carotenoids absorption maxima, than the WT (Figure 4B). Moreover, both overexpression strains showed lower carotenoid contents (Figure 4B) making them to appear bluish when compared to WT or STXC2 strains. These phenotypes were exacerbated in air bubbled cultures in which carbon availability is further reduced. Under these conditions, all strains showed a reduced growth rate (Figure 4C), although growth of WTOE and STXCOE strains was reduced more than that of WT and STXC2 strains (Figure 4C). Despite the STXC2 strain growth rate being very similar to the WT, it contained even higher carotenoids which gave a yellow appearance to the cultures (Figure 4D,E). Overexpression strains showed similar carotenoids contents that were lower than WT levels (Figure 4D). Furthermore, the STXC2 strain showed higher chlorophyll contents than other strains in both 1% CO_2_ (4.1 ± 0.3 µg chl OD_750nm_^−1^ for STXC2 vs 3.5 ± 0.5, 3.5 ± 0.2 and 3.4 ± 0.3 µg chl OD_750nm_^−1^ for WT, WTOE and STXCOE, respectively) and air bubbled conditions (3.83 ± 0.5 µg chl OD_750nm_^−1^ for STXC2 vs. 3.1 ± 0.3, 3.5 ± 0.3 and 3.5 ± 0.2 µg chl OD_750nm_^−1^ for WT, WTOE and STXCOE, respectively). All these results suggest that TrxC is involved in adaptation to low carbon conditions and that it could be mediated by regulating pigment content in *Synechocystis*.

### 3.4. Photosynthetic Characterization of TrxC Mutant Strains

**Figure 5:**
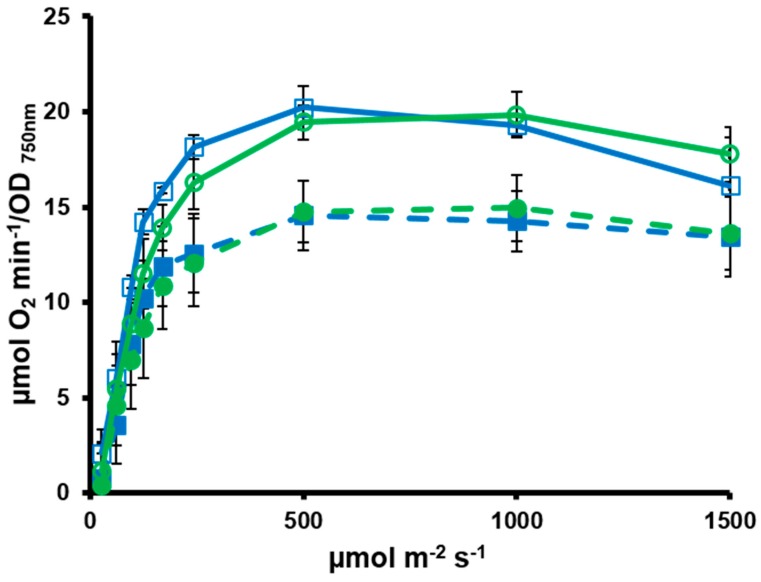
Overexpression of *trxC* causes lower photosynthetic efficiency. Oxygen evolution was measured in a Clark electrode at increasing light intensities in exponential growing cultures (OD_750nm_ = 0.5–1) of WT (□), STXC2 (○), WTOE (■) and STXCOE (●) grown in BG11 pH 7.5 at 180 μmol m^−2^ s^−1^ light intensity and bubbled with air.

To further characterize these strains, we determined different photosynthetic parameters using a Clark type oxygen electrode and DUAL-PAM 100 fluorimeter in air bubbled cultures. A light saturation curve was performed in exponentially growing cells of WT, WTOE, STXC2 and STXCOE in the oxygen electrode. WT and STXC2 showed similar light saturation curves saturating at around 500 µmol photons m^−2^ s^−1^ and reaching 20 µmol O_2_ min^−1^ per OD_750nm_ (Figure 5). Both overexpression strains also showed a similar behavior between them, saturating at the same light intensity of WT and STXC2 strains but reaching only 15 µmol O_2_ min^−1^ per OD_750nm_ (Figure 5). This lower photosynthetic capacity correlates with growth of these strains under this condition (Figure 4C). When photosynthesis was analyzed using a DUAL PAM100 fluorimeter, opposite results were obtained (Table 2). Fv’ in the dark-adapted state was higher for the overexpression strains than in WT and STXC2, indicating a higher fraction of open PSII centers in these strains. In contrast, Fv (measured in the presence of DCMU) was much more similar in all strains. These suggest that the amount of open PSII reaction centers is higher under physiological conditions (Fv’) but not when the photosynthetic electron flow is blocked (Fv). These data suggest that there are differences in the reduction state of plastoquinone pool. This is reinforced when NPQ (in dark adapted cells) is calculated as both overexpression strains showed lower NPQ. 

## 4. Discussion

Cyanobacteria contain a complex redox proteome which include four thioredoxin classes, at least three different types of thioredoxin reductases, four glutaredoxin classes and the GSH/glutathione reductase system [2,6,32]. Of the four thioredoxins, only TrxC is exclusively present in these groups of organisms and the fact that is present in most of them (with the exception of *Gloeobacteria* and marine *SynPro* clade) suggests that it has an important role in their cell physiology. Interestingly, the two groups in which TrxC is not present also lack FTR, Trx *x* and Trx *y* sequences in their genomes, indicating a reduction of their redox regulatory network. Although TrxC clearly belongs to the thioredoxin family, it shows an altered active site (WCGLCR) that is otherwise invariable for different thioredoxins from cyanobacteria, plants, bacteria or human. This sequence is missing the conserved P in the active site but two other prolines important for Trx structure are conserved in all TrxC sequences. The first proline conserved is five residues from the active site (P67 in Figure 1A) and the second one is from the cis-proline loop which also contains an adjacent threonine that is also conserved (positions 105 and 105 in Figure 1A) [1,33,34]. Other key residues in thioredoxins are also conserved such as phenylalanines in the N-terminal part of the sequence (F39 and F54 in Figure 1A) or an aspartate that is located opposite to the active site (D89 in Figure 1 A), although only one of the two conserved glycines in the C-terminal part of the protein are conserved (G112 in Figure 1A) [1]. The biochemical characterization of the protein has shown that *Synechocystis*’ TrxC is inactive in two classical thioredoxin activity assays (Figure 1B,C), in agreement with the data available for *Anabaena*’s TrxC that is unable to reduce OpcA [21]. Although there are no known thioredoxin that present a similar active site to the one in TrxC, site directed mutagenesis of the proline in the active site of *E. coli* Trx1 (P34H) or *Staphylococcus aureus* Trx (P31T or P31S) changed the redox potential of these proteins to more oxidizing [1,35,36,37]. This may be the reason that makes TrxC inactive in the classical thioredoxin assays although it is clearly not the only reason as the L32P mutant was also inactive (Figure 1B). Therefore, it will be worth determining redox potential and structure of this protein to clarify its function. Several other thioredoxins have been shown to be inactive in insulin reduction assays and function and targets of these proteins are not known [30,38]. Recently it has been shown that in plants Trx-fold proteins (TRXL1/2 and ACHT4) are involved in oxidizing, rather than in reducing, proteins during the night or low light conditions allowing to fine tune metabolism in response to changes in light availability [39,40]. Furthermore, DDOR was induced in both WTOE and STXCOE strains, that also showed elevated TrxC protein levels (Figure 2 and Figure 3), suggesting that these proteins might be functionally related. As DDOR is probably an oxidase [11] and changes in proline of the active site in thioredoxins (like the one present in TrxC) make them more oxidizing [1,37], it would be possible that DDOR-TrxC can function as a redox couple during stress in a similar way as the TRXL2/2cys-peroxiredoxin works in *Arabidopsis* [39].

The physiological characterisation of *trxC* mutants has shown that TrxC could be involved in adaptation to light and/or carbon availability because mutants in *trxC* showed a differential phenotype under conditions that change these two parameters. The STXC2 strain, although growing at similar rates to wild type, showed an increased carotenoid content in cultures bubbled with 1% CO_2_ or air (Figure 4), suggesting increased photoprotection. In contrast, both overexpression strains showed lower carotenoid contents, suggesting that TrxC somehow regulates pigment accumulation. The lower carotenoid content in the overexpression strains can also explain the lower photosynthetic activity exhibited by these strains at higher light intensities (Figure 4 and Figure 5) when carotenoids are important to maintain photosynthetic activity by preventing oxidative damage to the photosynthetic machinery [41]. It also explains their reduced growth rate under low carbon conditions in which light absorbed by the photosystems cannot be used as efficiently for CO_2_ fixation and therefore more Reactive oxygen species (ROS) are produced. In contrast, when photosynthesis was analyzed by DUAL-PAM 100, overexpression strains showed more open PSII reaction centers in the dark (Fv’_dark_/Fm’_dark_) than WT and STXC2 strains, and therefore more photosynthetic efficiency. When we measured the same parameter in the presence of DCMU, all strains showed a similar value indicating that the photosynthetic machinery is similar in all strains indicating that there is no difference in total photosynthetic machinery. The higher Fv’_dark_/Fm’_dark_ indicated a more oxidized plastoquinone pool. Several mechanisms can explain this, such as lower cyclic electron transport, higher Mehler-like reactions catalyzed by flavodiiron proteins or increased respiration [42] which will be seen as reduced oxygen evolution due to enhanced photoreduction. These could also explain the lower growth rate of these strains under this condition as they imply a drain of electrons from the photosynthetic electron transfer chain. Another explanation is that these strains contained more phycobilisome (PBS), which can be suggested by the apparent color changes of the overexpression strains that appear bluish. Nevertheless, whole cell spectra showed that the difference in color can be ascribed to changes in carotenoids and not in PBS (Figure 4B,D). In agreement with these data, a *trxC*^−^ mutant strain in *Anabaena* also showed altered pigment contents [20]. In this case it contained less chlorophyll and PBS together with less structured thylakoids membranes [20]. Furthermore, it also showed lower catalase activity and higher lipid peroxidation suggesting a redox imbalance [20], although whether the redox stress is caused by the photosynthetic defect or vice versa was not elucidated. Although our data shows that TrxC regulates pigment contents and photosynthetic activity, further characterization of the mutant strains is required to understand the molecular mechanism of these differences.

Finally, analysis of the genomic context has shown that *trxC* is adjacent to *nnrU* gene in many genomes (and is actually annotated as NnrU associated thioredoxin). NnrU is a membrane protein, and although its function is unknown, it is possible that it could functionally interact with TrxC. In *Nostocales trxC* is not only associated to *nnrU* but also to *ndhFM* genes which are part of NDH-1L complex involved in cyclic electron flow and respiration [43,44]. It is possible that some of the phenotypes observed such as slow growth in low carbon condition, lower photosynthetic activity or more oxidized plastoquinone pool in the dark can be ascribed to partially non-functional or non-regulated NDH-1L complex. In fact, *ndhF1* mutants in *Synechocystis* show a similar phenotype to WTOE and STXOE strains with lower oxygen-evolving activity but higher Fv’/Fm’ than the WT [45]. This is reinforced as *Anabaena trxC*^−^ mutant showed higher levels of NdhF1 protein and other electron transport proteins involved in cyclic electron transport [20]. All these data suggest that TrxC could modulate negatively NdhF1 (and therefore NDH-1L complex) activity and or assembly, although further experiments are needed to confirm this hypothesis. 

## 5. Conclusions

In summary, here we have characterized TrxC, an unusual thioredoxin that is present exclusively in cyanobacteria, and showed that it is inactive in classical thioredoxin assays. Furthermore, we have analyzed both *trxC* knockout and overexpression mutants and showed that these are affected in pigment composition, growth and photosynthetic activity, although the mechanisms remain unknown and will require further characterization of the mutant strains. 

## Figures and Tables

**Table 1:** *Synechocystis* strains used in this work.

Strain	Relevant Genotype	Mutated ORFS	Source
WT	*Synechocystis* sp PCC 6803	-	Lab collection
STXC2	*ΔtrxC::C.K1*	*sll1057*	[25]
WTOE	*glnN::PpetE:histrxC:Nat*	*slr0288*	This study
STXCOE	*ΔtrxC::C.K1 glnN::PpetE:histrxC:Nat*	*sll1057*, *slr0288*	This study

**Table 2:** Photosynthetic parameters calculated using a DUAL-PAM 100 fluorimeter from cultures grown as in Figure 4.

STRAIN	F_0_	Fm DCMU	Fv	Fv/Fm	Fm’ (Dark)	Fv’ (Dark)	Fv’/Fm’ (Dark)	NPQ (Dark)
WT	0.363 ± 0.028	0.563 ± 0.033	0.200	0.356	0.427 ± 0.031	0.064	0.150	0.319
WTOE	0.424 ± 0.049	0.617 ± 0.043	0.193	0.313	0.505 ± 0.044	0.080	0.160	0.223
STXC2	0.395 ± 0.066	0.597 ± 0.066	0.202	0.339	0.463 ± 0.076	0.069	0.148	0.288
STXC2OE	0.390 ± 0.028	0.601 ± 0.028	0.211	0.351	0.482 ± 0.027	0.091	0.190	0.248

## Supplementary Materials

The following are available online at http://www.mdpi.com/2076-3921/7/11/164/s1, Table S1: Oligonucleotides used in this work. Table S2: Conservation of *trxC* in different cyanobacteria.
