# Supplementary material for: Characterization of TrxC, an Atypical Thioredoxin Exclusively Present in Cyanobacteria
Source: Antioxidants (Basel). 2018 Nov 13;7(11):164. doi: 10.3390/antiox7110164 (PMC6262485; doi:10.3390/antiox7110164)
Supplement: Supplementary file 1 [file antioxidants-07-00164-s001.pdf]

Table S1. Oligonucleotides used in this work.

Oligo number	Oligo name	Oligo sequence
1	TrxCHIII	AAGCTTGGAATTGGTTGTTGTC
2	trxCXhoI	GTCGAGACAACCTGTACTAGCTT
50	glnN_check_F	ATGCAGGCCAGTCTTCCTAA
51	glnN_check_R	AAATGGCAGTGTCCAAGTCC
140	TrxC_NotI	AACCCGCGGCCGCCACTGGATGGTTCAGG GAGAG
141	TrxC_NdeI	AACATGCATATGCTAGCCGTTAACGAAGA C
155	trxC_L32P_Fw	TGGTGCGGACCATGTCACTTT
156	trxC_L32P_Rv	AAAGTGACATGGTCCGCACCA
186	TrxC_F_KO_check	CAACAAGGGATCAGTCCGCT
187	TrxC_R_KO_check	CGCCAATGTCTTGACCTTGG
192	Syn_TrxC_BamHI_NdeI	AGAGGATCCCATATGCTAGCCGTTAAC
193	SynTrxC_R_SalI	CGTCTGGTCGACTCAGGGAGAGAAAAG

Table S2. Conservation of *trxC* in different cyanobacteria.

order	trxC gene present
Chroococcidiopsidales	yes
Gloeobacteria	No
Gloeoemargaritales	yes
Nostocales	yes
Aphanizomenonaceae	yes
Chlorogloeopsidaceae	yes
Fortieaceae	yes
Hapalosiphonaceae	yes
Nostocaceae	yes
Rivulariaceae	yes
Scytonemataceae	yes
Tolypothrichaceae	yes
Oscillatoriophycideae	Yes
Pleurocapsales	yes
Spirulinales	yes
Synechococcales	yes
Acaryochloridaceae	yes
Chamaesiphonaceae	yes
Leptolyngbyaceae	yes
Merismopediaceae	yes
Prochloraceae	No
Pseudanabaenaceae	yes
Synechococcaceae	yes
